# Non-Parametric Statistical Thresholding for Sparse Magnetoencephalography Source Reconstructions

**DOI:** 10.3389/fnins.2012.00186

**Published:** 2012-12-26

**Authors:** Julia P. Owen, Kensuke Sekihara, Srikantan S. Nagarajan

**Affiliations:** ^1^Biomagnetic Imaging Laboratory, Department of Radiology and Biomedical Imaging, University of California San FranciscoSan Francisco, CA, USA; ^2^Joint Graduate Group in Bioengineering, University of California San Francisco/University of California BerkeleySan Francisco, CA, USA; ^3^Department of Systems Design and Engineering, Tokyo Metropolitan UniversityTokyo, Japan

**Keywords:** non-parametric statistics, sparse source reconstruction, magnetoencephalography, maximal statistic, non-invasive brain imaging

## Abstract

Uncovering brain activity from magnetoencephalography (MEG) data requires solving an ill-posed inverse problem, greatly confounded by noise, interference, and correlated sources. Sparse reconstruction algorithms, such as Champagne, show great promise in that they provide focal brain activations robust to these confounds. In this paper, we address the technical considerations of statistically thresholding brain images obtained from sparse reconstruction algorithms. The source power distribution of sparse algorithms makes this class of algorithms ill-suited to “conventional” techniques. We propose two non-parametric resampling methods hypothesized to be compatible with sparse algorithms. The first adapts the maximal statistic procedure to sparse reconstruction results and the second departs from the maximal statistic, putting forth a less stringent procedure that protects against spurious peaks. Simulated MEG data and three real data sets are utilized to demonstrate the efficacy of the proposed methods. Two sparse algorithms, Champagne and generalized minimum-current estimation (G-MCE), are compared to two non-sparse algorithms, a variant of minimum-norm estimation, sLORETA, and an adaptive beamformer. The results, in general, demonstrate that the already sparse images obtained from Champagne and G-MCE are further thresholded by both proposed statistical thresholding procedures. While non-sparse algorithms are thresholded by the maximal statistic procedure, they are not made sparse. The work presented here is one of the first attempts to address the problem of statistically thresholding sparse reconstructions, and aims to improve upon this already advantageous and powerful class of algorithm.

## Introduction

Magnetoencephalography (MEG) and electroencephalography (EEG) are powerful non-invasive neuroimaging technologies that can resolve brain activity on the order of a millisecond. Unlike brain imaging methods that directly measure correlates of brain activity, such as functional magnetic resonance imaging (fMRI) and positron emission tomography (PET), the neural activity at every location in the brain or “voxel” must be estimated from the surface magnetic or electric fields recorded with M/EEG. This estimation process is referred to as “source localization” and solving this ill-posed inverse problem is one of the greatest challenges to using M/EEG to elucidate neural activations. Major advances have been made in developing source localization algorithms, yet the statistical thresholding of the results obtained from these solutions remains an unresolved issue in the field.

Statistically thresholding non-invasive brain imaging data, in general, can be broken down into two steps: computing voxel-level statistics and image-level thresholding. In the voxel-level statistics step a test statistic is calculated for each voxel along with a corresponding *p*-value, the probability that the statistic value would exceed that which was observed under the null hypothesis. The method for obtaining the *p*-values can be either parametric or non-parametric. These *p*-values can then be thresholded, the image-level thresholding step, to a level at which the results are unlikely to have been observed by chance. Usually, results are accepted if they have either a 1 or 5% chance of occurring at random, corresponding to *p* < 0.01 or 0.05, respectively. In the case of brain imaging, there can be 5,000 to 100,000 voxels, which results in numerous voxel-level statistical tests occurring in parallel. Therefore, the risk of committing a Type I error, falsely identifying significant activity, is high. There are multiple methodologies to correct for this risk, or family wise error rate (FWER), including the Bonferroni (1935) correction, false discovery rate (FDR), both implemented in a step-up (Benjamini and Hochberg, 1995) and a step-down procedure (Benjamini and Liu, 1999), and applications of Gaussian random field theory (Nichols and Holmes, 2001). In addition to these corrections, which can be applied to parametric and non-parametric methods, the maximal statistic approach corrects for FWER in a non-parametric, resampling framework. A comprehensive review of these issues as they apply to neuroimaging can be found in Nichols and Holmes (2001).

Non-parametric permutation or resampling methods have been applied extensively to M/EEG data to find statistical thresholds for both single-subject brain activation maps and to detect group differences (Nichols and Holmes, 2001; Singh et al., 2003; Chau et al., 2004; Pantazis et al., 2005; Sekihara et al., 2005; Dalal et al., 2008). Many of the techniques described in these papers are developed, borrowed, or adapted from methods designed for fMRI/PET data. The M/EEG source localization algorithms used to reconstruct brain activity in these papers generally produce source images somewhat resembling those of fMRI in that they are diffuse and have a roughly Gaussian profile. Two such commonly used classes of algorithms are minimum-norm estimate (MNE; Hämäläinen and Ilmoniemi, 1994) and beamformers (Sekihara and Nagarajan, 2008). In recent years, sparse algorithms have gained traction in the M/EEG community. Sparse algorithms have a drastically different source power profile; the majority of voxels have zero or near-zero power and only a small fraction of voxels contain the power seen in the sensor recordings. Sparse methods, such as minimum-current estimate (MCE; Uutela et al., 1999), FOCUSS (Gorodnitsky and Rao, 1997), Champagne (Wipf et al., 2009, 2010; Owen et al., 2012), and other methods (Ding and He, 2008; Bolstad et al., 2009; Ou et al., 2009) have been demonstrated to have advantages over non-sparse algorithms. One of these advantages is that the brain images obtained are focal and often do not require further thresholding to make them interpretable. While these images might not require thresholding, there can be spurious peaks that are not functionally relevant and could be thresholded to obtain more useful images.

We seek to answer three questions. First, can non-parametric resampling-based statistical thresholding methods be applied to the inverse solution obtained from sparse algorithms? Second, can non-parametric statistical thresholding reject spurious peaks in the already sparse image? And third, can brain images obtained from non-sparse algorithms resemble the sparse maps through stringent thresholding? First we introduce a source localization procedure with unaveraged sensor data and two proposed non-parametric statistical thresholding techniques hypothesized to be compatible with sparse algorithms. The methods are applied to simulated data with three, five, or ten sources (at varying SNR levels) and three real MEG data sets consisting of one, two, and three principal brain sources. We focus on the performance of statistical thresholding of sparse images with Champagne and compare the results to another sparse method, a variant of MCE referred to as generalized MCE (G-MCE; Wipf et al., 2009), and to two non-sparse methods, minimum-variance adaptive beamforming (MVAB; Sekihara and Nagarajan, 2008) and sLORETA (SL; Pascual-Marqui, 2002), a variant of MNE similar to dSPM (Dale et al., 2000).

## Materials and Methods

### Source localization with unaveraged data

We performed source localization on the unaveraged sensor data, with each trial aligned to the stimulus, by choosing a time window of approximately 100 ms in the pre-stimulus period and a time window of approximately 200 ms in the post-stimulus period from a total of *N* trials, where *N* is always less than the number of trials collected. (The exact time windows and number of trials differed between the data sets and these parameters can be found in the sections below.) Then, we concatenated the pre-stimulus windows and the post-stimulus windows to form one long pre-stimulus period *B^pre^* and post-stimulus period *B^post^* consisting of *N* trials of data. The source localization algorithms, Champagne, sLORETA, and G-MCE, were run on *B^pre^* and *B^post^*. The theory and details of the implementation of the algorithms, including Champagne, can be found in Owen et al. (2012). All the source localization methods generate a spatial filter *w* such that:

(1) $$s_{r}\left(t\right)=w_{r}B^{post}\left(t\right).$$

where *r* is the voxel index and *t* are the time points in the post-stimulus period.

The source time courses *s_r_*(*t*) were averaged across trials *N* and the power map *P* in a given time window (*t*_2_ ≥ *t* ≥ *t*_1_) was calculated across voxels:

(2) $$P_{r}=\frac{1}{T}\overset{t_{2}}{\underset{t=t_{1}}{\sum }}\;{\left(\frac{1}{N}\overset{N}{\underset{n=1}{\sum }}\;s_{r}(\mathrm{n,t})\right)}^{2}$$

where *T* is the number of time points in the window and *t*_2_ ≥ *t* ≥ *t*_1_ were selected individually for each data set. These parameters are specified in the sections below.

### Non-parametric statistical thresholding

#### Maximal statistic

We employed a resampling method, similar to the one proposed in Sekihara et al. (2005), to obtain a non-parametric statistical threshold. Since the null hypothesis is that there is no signal source activity at each voxel location, we chose to generate our surrogate data sets by resampling the pre-stimulus data by randomly drawing *N* trials from the total trials available (greater than *N*). We chose *N* to be the same number of trials used for the source localization procedure described above. By resampling the pre-stimulus period, we avoid signal leakage introduced by the commonly used procedure of randomly exchanging pre- and post-stimulus periods. MEG data sets typically contain on the order of 100 trials. If we choose to draw *N* = 30 trials, then there will be 10030 possible surrogate data sets. Generating every possible surrogate distribution results in millions of distributions; as such, we chose to subsample the surrogates by randomly creating *M* = 1000 total surrogate data sets. To ensure normalization between the surrogate and the original data sets, we normalized the power of each surrogate to the power of the original sensor data. To do this, we first calculated the sensor power in the post-stimulus period of the original data across time and sensors and then we multiplied each surrogate data set by the ratio of the original post-stimulus power to the surrogate sensor power (also computed across time and sensors). This normalization creates more stability in the maximal statistic distribution, described below.

The spatial filter weights obtained from the source localization procedure were applied to each surrogate data set to obtain source time courses, which were averaged across trials to generate a trial-averaged time course for every voxel. For each surrogate, we can calculated the power, $P_{r}^{m}$ in the time window (*t*_2_ ≥ *t* ≥ *t*_1_) across voxels, generically referred to as $\Psi_{r}^{m}$.

**Figure 1:**
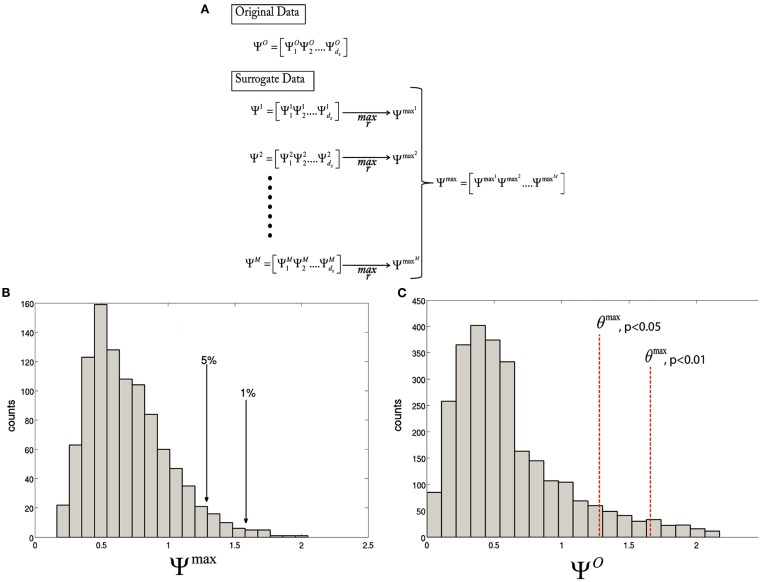
**Diagram illustrating the statistical thresholding procedure**. **(A)** The test statistic is calculated for every voxel for the original data,$\Psi_{r}^{O}$. Then, for each resampling of the data, $\Psi_{r}^{m}$ is computed. Finally, the maximum over *r* is taken to obtain $\Psi^{max^{m}}$. **(B)** A histogram of the maximal distribution, $\Psi^{max^{m}}$, with arrows pointing to the 1st and 5th percentiles, corresponding to *p* < 0.01 and 0.05, respectively. **(C)** A histogram of the original statistic, $\Psi_{r}^{O}$, with the *θ^max^*, *p* < 0.01 and *θ^max^*, *p* < 0.05, corresponding to the values obtained in **(B)**.

To employ the maximal statistic correction for both methods, we then took the maximum across voxels $\Psi_{r}^{m}$ from each surrogate:

$$\begin{array}{llll} \Psi^{max^{m}}=\underset{r}{\text{max}}\left(\Psi_{r}^{m}\right), & & & \text{(3)} \end{array}$$

and use the $\Psi^{max^{m}}$ to estimate the null distribution of Ψ*^O^*. Given a significance level of α, a statistical threshold, *θ^max^* can be set as the *c* + 1 largest member of $\Psi^{max^{m}}$, where *c* = *αM* and *c* is rounded down if not an integer. Ψ*^O^* can be thresholded by *θ^max^*, with a corresponding value for α. The statistical thresholding procedure for the maximal statistic are depicted in Figure 1. In this paper, we use maximal statistic thresholds at α = 1 and 5%, corresponding to *p* *<* 0.01 and 0.05, respectively.

#### Alternative to maximal statistic

**Figure 2:**
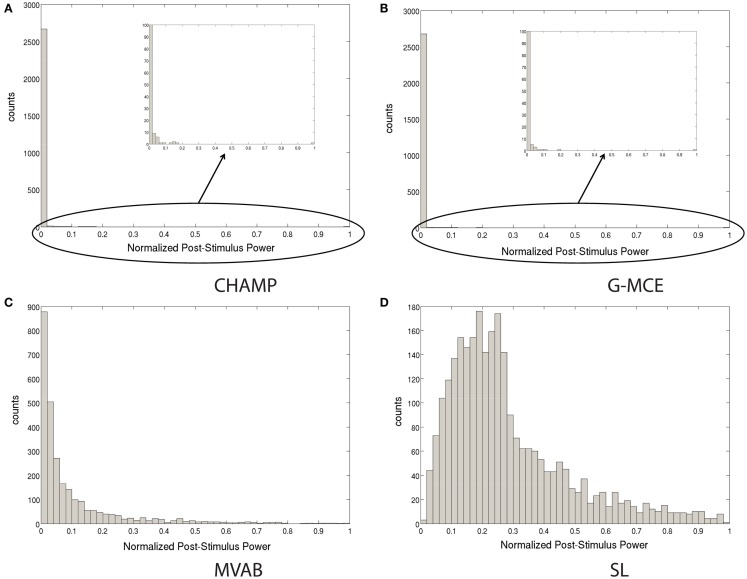
**Histograms of the post-stimulus power to illustrate the difference between sparse algorithms, Champagne (A) and G-MCE (B), and non-sparse algorithms, MVAB (C) and SL (D)**.

The widely used maximal statistic procedure was not designed with sparse algorithms in mind. In Figure 2, we plot the histogram of the source power for Champagne, G-MCE, MVAB, and SL obtained from a representative data set. In the sparsity profile of MVAB/SL as compared to Champagne/G-MCE, the histogram of the post-stimulus power values across voxels is drastically different in shape. SL/MVAB have a more or less smooth histogram, while Champagne/G-MCE have many voxels with little tono power and only a small subset with high power. The difference between the highest power value for Champagne and the second highest power value is large. And, even when we resample the pre-stimulus period to create surrogate data sets, this distribution of power values persists. If only the maximum statistic is saved for the null distribution, the threshold obtained can be driven by spurious voxels.

A less conservative approach than the maximal statistic is to save more than just the maximum statistical value from every surrogate data set. We have found that the maximal statistic can be driven by outliers; if there is one errant voxel (with high power) in each surrogate, the threshold obtained for Ψ*^O^* could be overly conservative. We propose saving the top *n*th percentile of the statistic values from each surrogate. The resulting distribution $\Psi^{n\%m}$ is used to estimate the null distribution of Ψ*^O^*. Just as with the maximal statistic, we can then obtain an alternative to the maximal statistic threshold, *θ^n%^*, by taking the *c* + 1 largest member of the distribution, where c=αñM, where *c* is rounded down if not an integer, ñ=(n∕100)V, and *V* is the total number of voxels. Then, Ψ*^O^* can be thresholded by *θ^n%^* with a corresponding value of *α*. We display results thresholded at α = 1 and 5%, corresponding to *p* < 0.01 and 0.05, respectively, for θ^1#x00025;^ and θ^5#x00025;^.

### Simulated MEG data

The simulated data in this paper was generated by simulating dipole sources. The brain volume was segmented into 8 mm voxels and a two-orientation (*d_c_* = 2) forward lead field (*L*) was calculated using a single spherical-shell model (Sarvas, 1987) implemented in NUTMEG (Dalal et al., 2004, 2011). One hundred trials were generated and the time course of each trial was partitioned into pre- and post-stimulus periods. The pre-stimulus period (200 samples) contained only noise and interfering brain activity. For the post-stimulus period (200 samples), the activity of interest, or the stimulus-evoked activity, was superimposed on the noise and interference present in the pre-stimulus period. The noise and interference activity (*E*) consisted of the resting-state sensor recordings collected from a human subject presumed to have only spontaneous neural activity and sensor noise. We tested 3, 5, and 10 sources; each source was seeded with a distinct time course of activity. We seeded the voxel locations with damped sinusoidal time courses (*S*). The intra-dipole (between dipole directions) and inter-source correlations were 0.5. The voxel activity was projected to the sensors through the lead field and the noise was added to achieve a signal to noise ratio (SNR) of −5, 0, 2, and 5 dB. We define SNR as:

(4) $$\text{SNIR}≜20\text{log}\frac{∥LS{∥}_{F}}{∥\varepsilon {∥}_{F}}.$$

where ||||F is the Frobenius norm.

The SNR levels were chosen to reflect a realistic range for single MEG trials. The simulated data had 275 sensor recordings.

The source localization was performed on the concatenated pre- and post-stimulus periods, as described above, on 30 of 100 data trials. We calculated the *A*′ metric (Snodgrass and Corwin, 1988) to assess the accuracy of the localization with each algorithm for each number of sources/SNR. The *A*′ metric estimates the area under the FROC curve for one hit rate and false-positive rate pair. The false-positive rate was calculated by dividing the number of false positive detected in each simulation by the maximum number of false positives found across all SNR levels for that number of sources, as in Owen et al. (2012). *A*′ ranges from 0 to 1, with a value of 1 indicating that all the sources were found and there were no false positives and a value of 0 indicating that only false positives were detected. To test the effectiveness of the maximal statistic and alternative thresholds, *A*′ was computed for each of the following statistical thresholds: *θ^max^*, *p* < 0.01 and 0.05; *θ*^1%^, *p* < 0.01 and 0.05; and *θ*^5%^, *p* < 0.05. (Empirically we found that *θ*^1%^, *p* < 0.05 and *θ*^5%^, *p* < 0.01 yield almost identical *A*′ results). We averaged the *A*′ over 10 runs for each number of sources/SNR pair. The *A*′ measure addresses whether a threshold is liberal enough to allow all true sources to survive, while also being stringent enough to reject false positives.

### Real MEG data sets

We selected three data sets based on the varying number of distinct brain activations expected in each. All MEG data was acquired in the Biomagnetic Imaging Laboratory at UCSF with a 275-channel CTF Omega 2000 whole-head MEG system from VSM MedTech (Coquitlam, BC, Canada) with a 1200 Hz sampling rate. As with the simulated data, the lead field for each subject was calculated in NUTMEG using a single-sphere head model (two-orientation lead field) and an 8 mm voxel grid. The data were digitally filtered from 1 to 160 Hz to remove artifacts and the DC offset was removed. The data sets were used in a performance evaluation paper of Champagne (Owen et al., 2012); in this previous work Champagne and the other algorithms were applied to averaged sensor data.

#### Single source: somatosensory evoked field

We used a somatosensory evoked field (SEF) data set. The stimulation is administered by air puffs with a pseudorandom interstimulus interval of 450–500 ms. For the pre-stimulus period, we took the window of data between −100 and −5 ms from each trial and for the post-stimulus period, we took the window between 5 and 200 ms, where 0 ms is the onset of the stimulus. We used the first 10 trials of data of 252 trails. We calculated the source power in the window between 40 and 80 ms and applied the statistical thresholding procedure. For this paradigm, we expect to localize one principal source in the contralateral somatosensory cortex.

#### Dual sources: auditory evoked field

We analyzed an auditory evoked field (AEF) data set for which the subject was presented single 600 ms duration tones (1 kHz) binaurally. We concatenated the first 35 out of 116 trials for this data set, choosing the window from −90 to −5 ms as the pre-stimulus period and the window from 5 to 200 ms as the post-stimulus period from each trial. We then calculated the power in the window around the M100, the auditory response, from 90 to 120 ms. For this data set, it is expected that we will localize bilateral auditory responses in primary auditory cortex.

#### Multiple sources: audio-visual task

We analyzed a data set designed to examine the integration of auditory and visual information. We presented single 35 ms duration tones (1 kHz) simultaneous to a visual stimulus. The visual stimulus consisted of a white cross at the center of a black monitor screen. The pre-stimulus period was selected to be the window from −100 to −5 ms and the post-stimulus window was taken to be 5–250 ms, where 0 ms is the onset of the simultaneous tone and visual stimulus. We concatenated the pre-stimulus and post-stimulus periods for the first 30 out of 97 trials. Then we computed the power in two windows, from 80 to 140 ms to capture the auditory activation and 100–180 ms to capture the visual activation. We applied the thresholding procedure to the auditory response and the visual response, separately. This data set is the most complex; we expect to localize two auditory sources in bilateral primary auditory cortex and at least one visual source in primary visual cortex.

## Results

### Simulated MEG data

**Figure 3:**
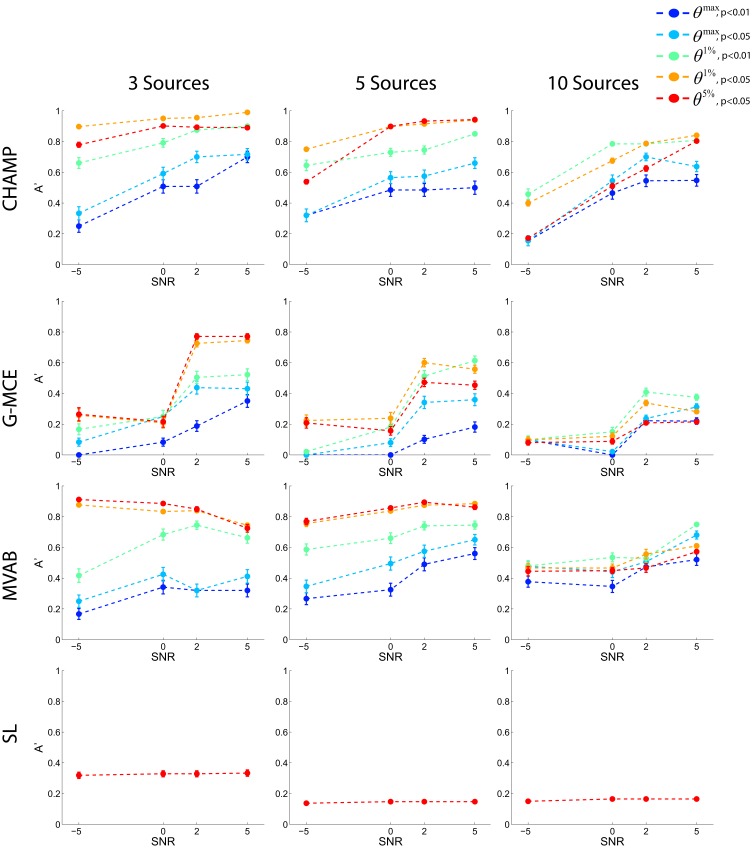
**Results with simulated data**. Data was generated with 3, 5, or 10 sources at SNR levels of −5, 0, 2, and 5 dB. Source localization was performed with CHAMP, G-MCE, MVAB, and SL using 30 trials of data. The *A*′ metric was used to quantify the localization and was averaged over 10 runs and each point is the mean *A*′ with a SE bar.

In Figure 3, *A*′ is plotted for each number of dipoles (columns of the figure) and SNR level across the 5 statistical thresholds: two maximal statistic thresholds, *θ^max^*, *p* < 0.01 and 0.05, and three for the alternative to the maximal statistic, the *n*th percentile thresholds, *θ*^1%^, *p* < 0.01 and 0.05, and *θ*^5%^, *p* < 0.05. Each point is an average across 10 runs and we plot the average and SE bars. The results from Champagne (first row) demonstrate that the *θ*^1%^, *p* < 0.05 and *θ*^5%^, *p* < 0.05 thresholds produce the highest average *A*′ values for the 3 and 5 source simulations. At 10 sources, more stringent thresholds, *θ*^1%^, *p* < 0.01 and *θ*^1%^, *p* < 0.05, produce the best results. The maximal statistic thresholds, *θ^max^*, *p* < 0.01 and 0.05, produce *A*′ values that underestimate the localization accuracy. The results with MCE (second row) demonstrate that the maximal statistic thresholds are overly stringent. The alternative to the maximal statistic maximize *A*′ for 3 and 5 sources at higher SNR levels, but with 10 sources and low SNR, MCE has difficultly localizing the sources as reflected by the *A*′ values. The *A*′ results from MVAB (third row) are similar to those obtained with Champagne; the *θ*^1%^, *p* < 0.05 and *θ*^5%^, *p* < 0.05 thresholds produce the highest average *A*′ values for 3 and 5 sources. However, with 10 sources, all thresholds produce similar *A*′ values and the localization is poor. The localization with SL (fourth row) reveal that this algorithm is not able to localize multiple sources in these simulated data sets. Generally, SL was able to localize only 1 source at all source numbers and SNR levels. The different levels of statistical thresholding produce identical *A*′ values as more stringent thresholding does not salvage the poor localization. We plot only the *A*′ results with the most liberal threshold *θ*^5%^, *p* < 0.05 for SL.

Overall, these simulations demonstrate that the maximal statistic is overly conservative for sparse reconstructions and the alternative to the maximal statistic thresholds provide higher average *A*′ values. Give these results, we investigate two maximal statistic thresholds, *θ^max^*, *p* < 0.01 and 0.05, and two alternative to the maximal statistic thresholds, *θ*^1%^, *p* < 0.05 and *θ*^5%^, *p* < 0.05, on the real MEG data sets.

### Real MEG data

We present the localization results with unaveraged data for three data sets, somatosensory evoked field (SEF), auditory evoked field (AEF), and audio-visual (AV) data sets. We ran Champagne on these data sets and compared the performance to G-MCE, SL, and MVAB. For all the overlays on the MRI presented here, we show the coronal (and axial) section that intersects the maximum voxel for the time window being investigated. We applied the maximal statistic thresholding procedure to the three data sets to investigate the effectiveness of our resampling procedure for sparse algorithms. We also compare these results to the results obtained from the alternative to the maximal statistic procedure and provide the thresholds expressed as a percentage of the maximum voxel power in the image.

#### Single source: somatosensory evoked field

**Figure 4:**
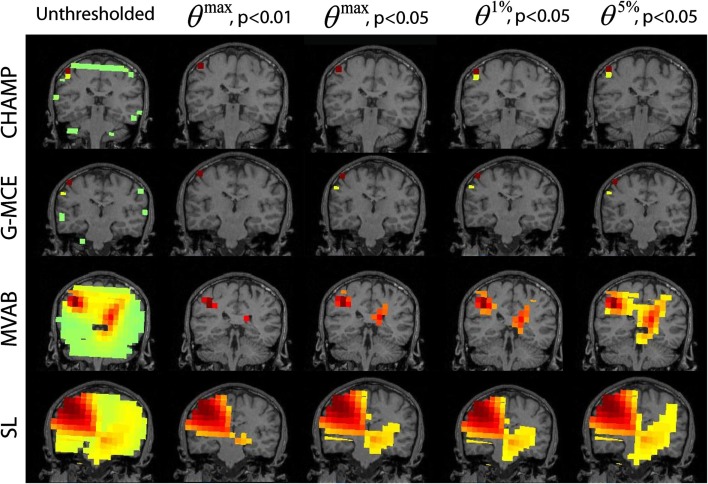
**Somatosensory (SEF) data: the source localization was performed on the first 10 trails of data**. The unthresholded post-stimulus power values in the window from 40 to 80 ms are shown in the first column (coronal slice). The power is thresholded with the maximal statistic: *θ^max^*, *p* < 0.01 and 0.05 and with the alternative to the maximal statistic thresholds: *θ*^1%^, *p* < 0.05, and *θ*^5%^, *p* < 0.05.

In Figure 4, we present the unthresholded source power results along with the thresholded results for *θ^max^*, *p* < 0.01 and 0.05 and *θ*^1%^, *p* < 0.05, and *θ*^5%^, *p* < 0.05 approach for all algorithms, Champagne, G-MCE, MVAB, and SL. The unthresholded results from Champagne demonstrate that it is able to localize the contralateral somatosensory cortex, but there are voxels in functionally irrelevant areas that are not pruned. Thresholding at all confidence levels cleans up the source power image. The maximal statistic thresholds leave only the source in the contralateral somatosensory cortex. As compared to the maximal statistic threshold, the alternative to the maximal statistic thresholds are less stringent and allow a second contralateral voxel to survive as well as an activation in the ipsilateral somatosensory cortex to pass the significance threshold (not visible in the coronal slice shown). The results from G-MCE are similar; the unthresholded power image shows that there is a source in somatosensory cortex, but there are also non-zero voxels in other brain areas. Thresholding at *θ^max^*, *p* < 0.01 leaves only the source in somatosensory cortex, and thresholding at less stringent levels reveals another source nearby. The unthresholded results for MVAB and SL show that there is a peak in the contralateral somatosensory cortex and the thresholding at all levels cleans up the images to some degree. All threshold levels remove more voxels for MVAB than SL, and the *θ^max^*, *p* < 0.01 level with MVAB has similar sparsity to Champagne and G-MCE. These thresholds expressed as a percent of the maximum voxel power for *θ^max^*, *p* < 0.01 and 0.05, *θ*^1%^, *p* < 0.05, and *θ*^5%^, *p* < 0.05, respectively, are: Champagne 43/34/13/5%, MCE 17/14/4/1%, MVAB 50/39/38/29%, and SL 33/25/23/20%.

#### Dual sources: auditory evoked field

**Figure 5:**
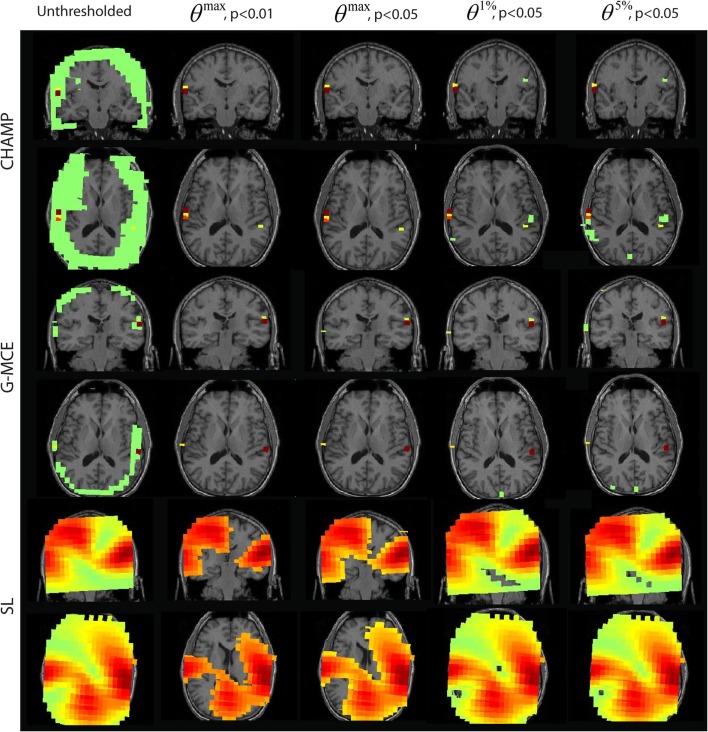
**Auditory evoked field (AEF) data: the source localization was performed on the first 30 trials of AEF data**. The unthresholded post-stimulus power values in the window from 90 to 120 ms are shown in the first column (coronal slice). The power is thresholded with the maximal statistic: *θ^max^*, *p* < 0.01 and 0.05 and with the alternative to the maximal statistic thresholds: *θ*^1%^, *p* < 0.05, and *θ*^5%^, *p* < 0.05.

The results from the AEF data are shown in Figure 5. The first column displays the unthresholded results from the unaveraged data for Champagne, G-MCE, and SL. All three algorithms show bilateral activity in the time window around the auditory response. For Champagne, the thresholded results for both levels of *θ^max^* are the same, leaving the bilateral auditory activity (the right activation can be seen in the axial slice). The alternative to the maximal statistic thresholds allow a larger cluster of voxels in auditory cortex to pass to significance, but *θ*^5%^, *p* < 0.05 allows a weak source in visual cortex to survive. G-MCE also localizes bilateral activity (the left activation can be seen in the axial slice) and the maximal statistical threshold at both levels, like Champagne, maintains the bilateral auditory voxels. The alternative to the maximal statistic thresholds do not augment the auditory activity, but rather allow voxels in visual cortex to pass to significance. The statistical thresholding for SL is still quite liberal even at *θ^max^*, *p* < 0.01 and the thresholding at this stringent level does not provide focal activations. The localization was not successful with MVAB (results not shown), so we did not perform the thresholding on these results. These thresholds expressed as a percent of the maximum voxel power for *θ^max^*, *p* < 0.01 and 0.05, *θ*^1%^, *p* < 0.05, and *θ*^5%^, *p* < 0.05, respectively, are: Champagne 5/3/0.5/0.1%, MCE 11/11/2/0.6%, and SL 50/27/5/3%.

#### Multiple sources: audio-visual task

**Figure 6:**
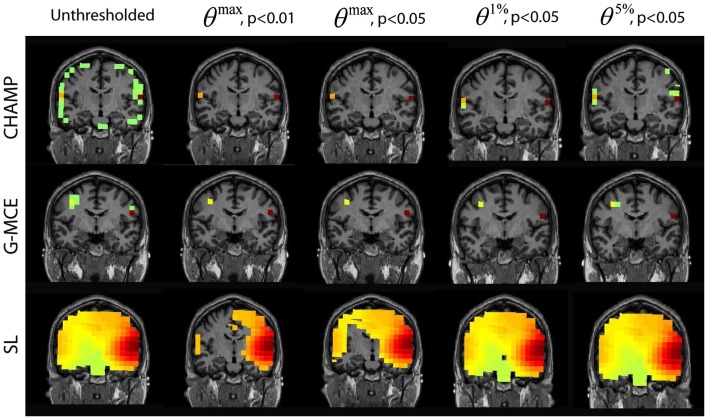
**AV data: the unthresholded post-stimulus power values in the window from 80 to 140 ms are shown in the first column (coronal slice), from source location on the first 30 trails of data**. The power is thresholded with the maximal statistic: 0*^max^*, *p* < 0.01 and 0.05 and with the alternative to the maximal statistic thresholds: *θ*^1%^, *p* < 0.05, and *θ*^5%^, *p* < 0.05.

The results for the auditory response of the audio-visual task are provided in Figure 6. For Champagne, the unthresholded post-stimulus power values are shown in the first column. We found that the *θ^max^* thresholds were sufficient to clean up the post-stimulus power maps, but not overly stringent; both auditory cortical sources remained after thresholding at all levels. Thresholding with *θ*^1%^, *p* < 0.05, and *θ*^5%^, *p* < 0.05 provides for a larger cluster of auditory voxels, but also some potential false positives with *θ*^5%^, *p* < 0.05. For G-MCE, the localization results show bilateral activity (the left source is dorsal to auditory cortex) and we found the amount of thresholding to be similar with all thresholds tested. SL is able to localize bilateral activity that is diffuse. The thresholding at *θ^max^*, *p* < 0.01 allows for distinguishing the left and right activations, although the right activation is still heavily biased. The less stringent thresholds do not create separation between the auditory activations. The localization for MVAB was unsuccessful (results not shown) and therefore we did not perform the thresholding procedure for MVAB. These thresholds expressed as a percent of the maximum voxel power for *θ^max^*, *p* < 0.01 and 0.05, *θ*^1%^, *p* < 0.05, and *θ*^5%^, *p* < 0.05, respectively, are: Champagne 36/16/4/3%, MCE 6/4/4/0.2%, and SL 32/23/9/5%.

**Figure 7:**
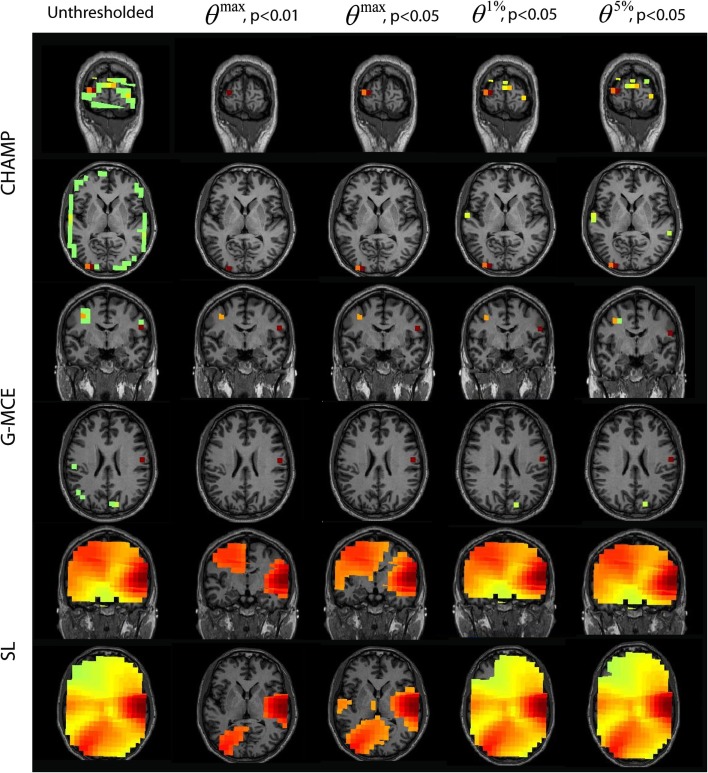
**AV data: the unthresholded post-stimulus power values in the window from 100 to 180 ms are shown in the first column (coronal slice), from source location on the first 30 trails of data**. The power is thresholded with the maximal statistic: *θ^max^*, *p* < 0.01 and 0.05 and with the alternative to the maximal statistic thresholds: *θ*^1%^, *p* < 0.05, and *θ*^5%^, *p* < 0.05.

The results for the visual localization with Champagne, G-MCE, and SL are presented in Figure 7. Champagne is able to localize visual activity in this time window; thresholding with *θ^max^* allows activation in one visual area to pass to significance. Compared to the maximal statistic threshold, the alternative to the maximal statistic thresholds are less stringent and allow more of the visual activity present in the unthresholded map to survive, although voxels in auditory cortex also survive at this more liberal significance level. The unthresholded results from G-MCE show that there are activations in auditory areas (as in the 80–140 ms time window) and there is activation in the visual cortex, but these voxels do not have the maximum power in the time window (different from Champagne). With the maximal statistic, only the right auditory source passes to significance at all levels and the visual activations are thresholded out. With the alternative to the maximal statistic thresholds, a voxel in visual cortex is preserved. SL also shows both visual and auditory activations in the unthresholded maps. At *θ^max^*, *p* < 0.01 and 0.05, the visual activation is distinguished from the auditory activation and *θ*^1%^, *p* < 0.05, and *θ*^5%^, *p* < 0.05 do not effectively threshold the image. These thresholds expressed as a percent of the maximum voxel power for *θ^max^*, *p* < 0.01 and 0.05, *θ*^1%^, *p* < 0.05, and *θ*^5%^, *p* < 0.05, respectively, are: Champagne 48/40/13/5%, MCE 12/10/9/0.4%, and SL 57/43/10/7%.

## Discussion

In this paper, we have demonstrated the application of two novel methods to statistically threshold single-subject brain-activity maps obtained from sparse algorithms. These methods are specifically tailored for sparse algorithms given the different source power distribution seen with this class of algorithm. We address three central questions in this investigation using real and simulated MEG data. First, we show that non-parametric statistical thresholding can be applied to the source estimates from sparse algorithms. Second, when applied to Champagne’s activation maps, these thresholding methods are able to produce statistical thresholds that preserve functionally relevant activity, while removing spurious voxels that do not get pruned away during source localization. Similar thresholding effects are observed with G-MCE, another sparse algorithm. Third, we find that statistical thresholding does not always remedy poor or diffuse source reconstruction. The MVAB results on the simulated and SEF data benefit from the statistical thresholding, but MVAB is unable to localize functionally relevant brain activity in the AEF or AV data sets due to the correlated sources in the data. This is a known drawback to beamformers (Sekihara and Nagarajan, 2008). However, SL is not able to localize more than one source in the multisource simulations, thus strict statistical thresholding does not provide recovery for the algorithm. SL is more successful with the real data and is able to localize functionally relevant brain activity in the real MEG data, but the unthresholded activations are overly diffuse. Even after thresholding at *θ^max^*, *p* < 0.01, the brain maps are often still diffuse and difficult to interpret, leading to the conclusion that sparse-like solutions cannot always be obtained with stringent statistical thresholding of a non-sparse algorithm.

The maximal statistic procedure, as applied to sparse algorithms, can be overly stringent. We demonstrate this with the simulation study and some of the real MEG data sets, motivating the comparison of the thresholds derived from the maximal statistic to those saving the top 1st and 5th percentiles. The alternative methods proposed retain some of the properties of the maximal statistic, while balancing hits and false positives. The benefit of these thresholds is most exemplified in the results with simulated data. The *A*′ metric values for the alternative to the maximal statistic reflect a maximization of hits, while minimizing false alarms for the majority of number of source/SNR pairs for Champagne, G-MCE, and MVAB. With the real data sets, saving more than just the maximum from each surrogate protects the threshold from the spurious, high-powered voxels, which are more prevalent in sparse algorithms and retains multiple voxels in the final thresholded image.

The method of statistical thresholding developed here diverges from conventional methods in the literature in two major ways. First we do not generate the surrogate data sets by exchanging the pre- and post-stimulus periods of randomly chosen trials, as in Pantazis et al. (2005). In Pantazis et al. (2005), a non-adaptive method was used to localize the sources. Champagne is an adaptive method, meaning the weights are dependent on the data. In contrast, non-adaptive methods, such as SL and other minimum-norm algorithms, do not factor in the data when calculating the weights. The adaptive and sparse nature of Champagne makes the conventional method of generating surrogates problematic. Champagne prunes the majority of voxels to be zero. When these sparse weights are then applied to new data, the locations in the brain where there is non-zero activity is highly constrained, see Figure 2. If the surrogates are generated by switching the pre- and post-stimulus period, the surrogate post-stimulus periods will contain some of signal in the original post-stimulus window used to calculate the weights. This has more of an effect on Champagne and other adaptive methods than it does on the non-adaptive methods. When using only pre-stimulus data for the surrogates, we are assessing the source power obtained on data that we assume has no signal of interest, which is the assumption under the null hypothesis. Given that the surrogate data sets have the same sensor power as the original data and the weights are fixed across all surrogates, resampling the pre-stimulus data does not underestimate the source power distribution for the surrogates.

The second point of divergence is that we use the post-stimulus power as opposed to a pseudo *t*-value (or other statistic). When we apply Champagne’s sparse weights to the pre-stimulus period in order to obtain an estimate for the variance, used in the pseudo *t*-value calculation, we only obtain non-zero variance in a small subset of the voxels. Usually the variance is pooled across neighboring voxels to protect from spurious values driving the *t*-values, but in Champagne’s case, with such focal activations, pooling the variance does not have a smoothing effect. Thus, we found it was more stable to use the post-stimulus power values as our measure. It should be noted that Champagne subtracts the baseline from the post-stimulus source estimates and thereby the power values obtained are effectively the subtraction of the pre- and post-stimulus power.

We found that the method we developed (for both the maximal and alternative to the maximal statistic) is less stringent than the method whereby the surrogates are generated by pre- and post-stimulus switching and a pseudo *t*-value calculation is used; when we apply this “conventional” method to the real data sets, the activations obtained from Champagne only had the voxel with maximum power passed to significance, even at very liberal threshold of *p* < 0.10. Conversely, when the conventional thresholding procedure is applied to activations obtained from SL and MVAB, we did not observe that the diffuse activations were made to be more focal than with our proposed methods.

Champagne and other sparse methods come close to providing inherently thresholded maps of brain activations, but as demonstrated here, there can be spurious non-zero voxels; explicitly integrating statistical thresholding into the source localization procedure is an avenue we plan to investigate in the future.

## Conclusion

In this paper, we explore some of the technical considerations of statistically thresholding sparse source reconstructions. We find that the “conventional” maximal statistic procedure is often overly stringent when applied to sparse images, thus motivating the two proposed statistical thresholding methods presented in this paper. These two methods reject spurious peaks while optimizing the hit rate versus false-positive rate in the simulated data and keeping functionally relevant activations in the sparse reconstructions of three real MEG data sets. This work is one of the first to look at the statistical thresholding of brain images obtained from sparse reconstruction algorithms and will improve the efficacy of these already powerful algorithms.

## Conflict of Interest Statement

The authors declare that the research was conducted in the absence of any commercial or financial relationships that could be construed as a potential conflict of interest.
